# A Comprehensive Approach to Assess *Arabidopsis* Survival Phenotype in Water-Limited Condition Using a Non-invasive High-Throughput Phenomics Platform

**DOI:** 10.3389/fpls.2015.01101

**Published:** 2015-12-15

**Authors:** Emilio Vello, Akiko Tomita, Amadou Oury Diallo, Thomas E. Bureau

**Affiliations:** Department of Biology, McGill University, MontrealQC, Canada

**Keywords:** drought, water limited, abiotic stress, phenotype, phenomics, near infrared, high throughput, images

## Abstract

With the rapid rise in global population and the challenges caused by climate changes, the maximization of plant productivity and the development of sustainable agriculture strategies are vital for food security. One of the resources more affected in this new environment will be the limitation of water. In this study, we describe the use of non-invasive technologies exploiting sensors for visible, fluorescent, and near-infrared lights to accurately screen survival phenotypes in *Arabidopsis thaliana* exposed to water-limited conditions. We implemented two drought protocols and a robust analysis methodology that enabled us to clearly assess the wilting or dryness status of the plants at different time points using a phenomics platform. In conclusion, our approach has shown to be very accurate and suitable for experiments where hundred of samples have to be screened making a manual evaluation unthinkable. This approach can be used not only in functional genomics studies but also in agricultural applications.

## Introduction

Climate changes and environmental pollution will have a significant impact in the food production worldwide. The increase of global temperature will cause more frequent drought events (Prasch and Sonnewald, 2013) defined as soil water deficit or low water availability (Harb et al., 2010). Moreover, a persistent expansion of aridity has been observed since the middle of the 20th century and this process will continue according to current projection models (Dai, 2011). In some areas where crop yield reduction is predicted, major improvements in plant breeding programs and agricultural technology have to be developed (Jones and Thornton, 2003).

The use of different phenomics technologies in plants is a key element to improve our knowledge of the genotype–phenotype association of desired agricultural traits (Neilson et al., 2015) such as the response to water deficit. Some of these methods have been taken from medical applications as is in the case of high-resolutions X-ray computed tomography. This has shown to be an excellent tool to analyze the development of root system at high resolution in a non-destructive way (Lontoc-Roy et al., 2005). Other methodologies have been born specifically for plant phenotyping such as the Scanalyzer HTS developed by LemanTec (LemnaTec GmbH, Wuerselen, Germany) to scan small plants using a variety of wavelength cameras. Worldwide, prestigious universities and research institutes have acquired these technologies such as the CT scanning laboratory for agricultural and environmental research and the McGill Plant Phenomics Platform (MP3) at McGill University (Canada), the Australian Plant Phenomics Facility at the University of Adelaide (Australia) and the Arkansas Center for Plant-Powered Production at Arkansas University (USA).

In addition to the availability of the homozygous genome-wide knockout lines (Furbank and Tester, 2011), *Arabidopsis* is part of the mustard family (*Brassicaceae*; Haudry et al., 2013) to which economically relevant crops belong such as the edible canola oil, the cabbage vegetables or the biofuel candidate *Camelina sativa* for which the overexpression of the *Arabidopsis* MYB96 has conferred a drought resistance phenotype (Lee et al., 2014). This coupled with its small size and short life cycle (Lack and Evans, 2001) makes of *Arabidopsis* an excellent model organism to explore with the Scanalyzer HTS (Lemnatec GmbH, Wuerselen, Germany).

Despite the progress achieved in methods to detect genotype–phenotype association and quantify plant phenotypes at high resolution (Klukas et al., 2014), there are still limitations. The development of plant growth protocols, new image analysis algorithms and statistical pipelines is essential to exploit the full potential of these phenomics platforms. In this paper, we present a comprehensive approach to assess drought stress survival experiments using the advantages of our phenomic platform. This method is not limited to a specific protocol and it can be easily implemented into other high-throughput phenotyping facilities.

## Materials and Methods

### Plant Lines

Two *Arabidopsis thaliana* drought responsive genes were used in this assay: *gtl1–5* (SALK_044308C: AT1G33240), and *drs1* (SALK_149366C: AT1G80710) in addition to ecotype Col-0 as wild type (WT). In previous studies, *gtl1–5* mutants have shown a resistant phenotype (Yoo et al., 2010) and *drs1* mutants a sensitive phenotype (Lee et al., 2010).

### Growth Conditions

Two different protocols named in this work “pot protocol” and “pellet protocol” have been used.

#### “Pot Protocol”

**FIGURE 1:**
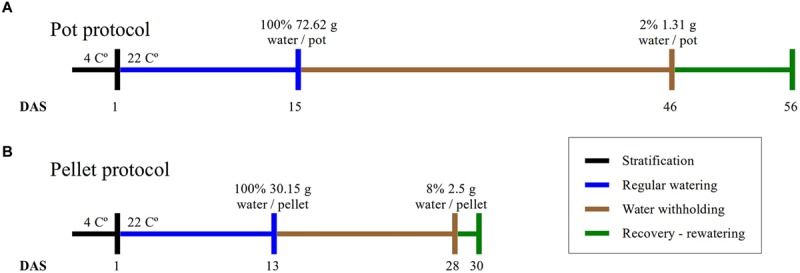
**Experimental protocols.** The duration of the four phases of the experiments and the absolute amount of water at the beginning and end of the water-withholding or water deficit period for **(A)** the “pot protocol” and **(B)** the “pellet protocol”.

Plants were grown in 2 1/2 square inch pots containing 20 g of 2:2:1 mixture of vermiculite, perlite, and peat moss (Cheng et al., 2013). After stratifying in the dark for 3 days at 4°C, pots were transferred to a growth chamber (Conviron TC30) at 22°C, 16/8-h light/dark photoperiod, 70% relative humidity and a light intensity of 135 mmol m^-2^ s^-1^. A number of 10 pots with WT, 10 pots with mutant *gtl1–5*, 10 pots with mutant *drs1* and 2 pots with soil only to control evaporation were put in each tray with a basket. The trays were sitting in an inverted basket for aeration purpose and black mulch (Fabricville, Canada; felt fabric Z048-BLK) was added on top of every pot. This tray configuration was used for the water-limited group and for the control or well-watered group. The distribution of the pots in each tray was regularly randomized using a computer algorithm as suggested by Skirycz et al. (2011) to minimize growth chamber effects. Here, this process has been done every 3–4 days and has taken 8 min per tray. The fill capacity of the pots was calculated by weighing the individual components, empty pots, mulch, pots with dry soil and 90 min after watering. Pots were weighed regularly during the experiment. After the appearance of the fourth leaf in over 60% of the plants, water was withheld for the drought or water-limited group until the plants exhibited lethal effects of dehydration or clear symptoms of wilting (Skirycz et al., 2011) after which the plants were rewatered (**Figure 1**). The pots (in the pot and in the mulch) and the trays (at side) had been labeled with barcodes to identify and trace the seedlings at each step of the experiment including the data analysis and the management of the randomization process.

#### “Pellet Protocol”

The plants were grown in Jiffy-7 pellets (Skirycz et al., 2011) inside basket-pots with diameter of 5 cm inserted in trays with spots separated by 1 mm. The growing conditions are the same as the “pot protocol” but the growth chamber (Conviron A1000). A number of eight pellets for each line including the WT and three “soil only” were put in each one of the two trays (one for the well-watered group and other for the water-limited group). Mulch has not been used and the fill capacity was calculated similarly as the previous protocol. A blue label for identification purpose was added and the samples were also randomized using a computer algorithm. The beginning and the end of the water-limited period have been done as described in the “pot protocol” (**Figure 1**).

### Data Acquisition

A customized version of the LemnaTec Scanalyzer High-Throughput Screening (HTS; LemnaTec GmbH, Wuerselen, Germany) installed at the McGill Plant Phenomics Platform (MP3^1^) was used to carry out the image acquisition. The unit has a robotic arm that holds sensors or cameras and moves to different positions inside the measurement cabinet. In this study, images were taken with the visible light camera piA2400-17gc (VIS), 2454 × 2056 pixels, the fluorescent light camera scA1400-17gc (FLUO), 1390 × 1038 pixels and the near infrared camera NIR-300PGE (NIR), 320 × 254 pixels. Every plant was imaged regularly during the experiments. The intensity of the NIR is measured from 0 to 255.

### Extraction of the Digital Plant and its Features

**FIGURE 2:**
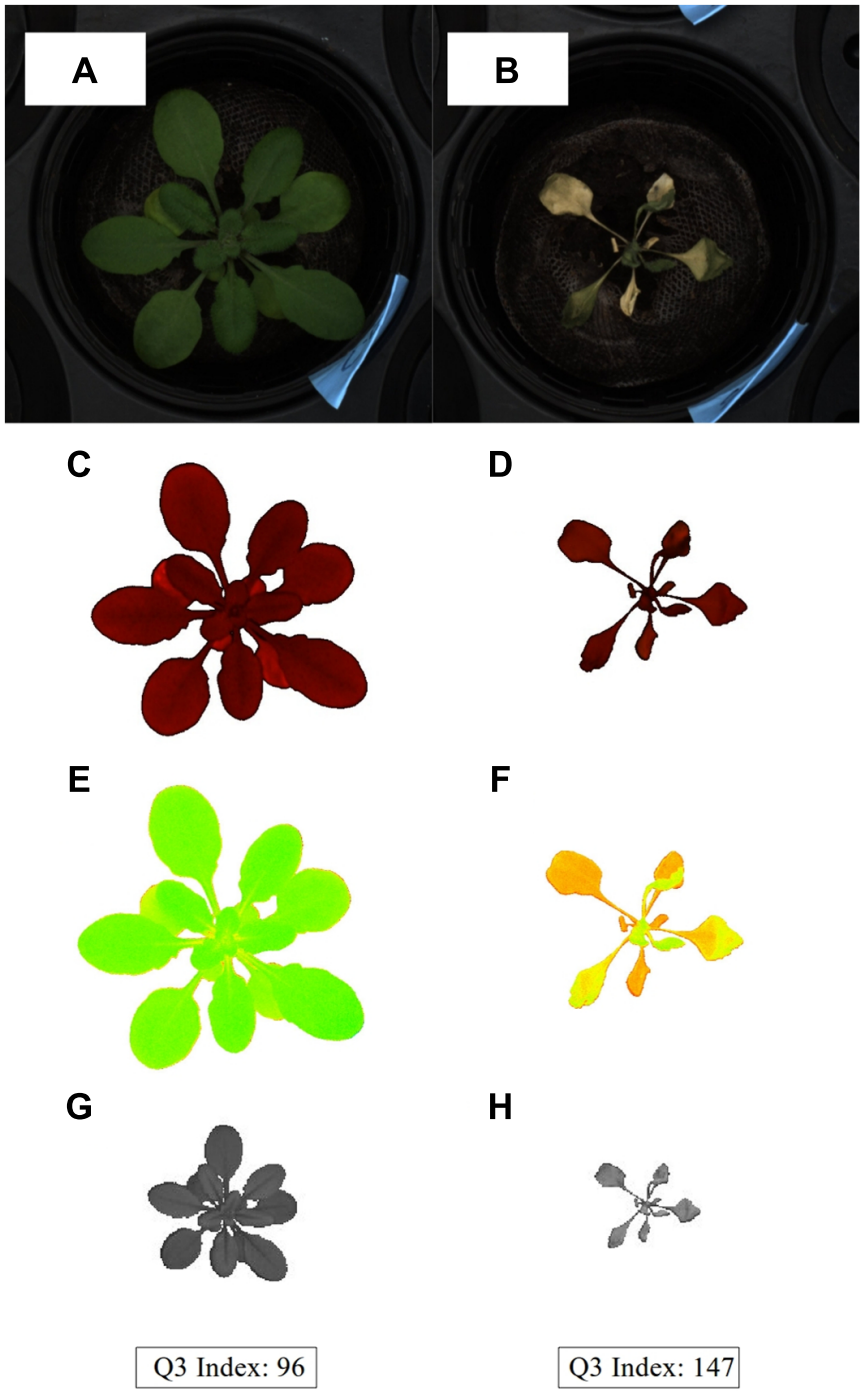
**Treatment phenotypes.** A sample from each treatment group **(A,C,E,G)** well-watered and **(B,D,F,H)** water-limited (Mutant line *drs1*, “pellet protocol”). Row images from VIS camera **(A,B)**. “Digital plants” from FLUO after segmentation process **(C,D)**. Color representation of the plants after color classification **(E,F)**. NIR “digital plants” with the indexes based on the third quartile (Q_3_) of the intensity histogram **(G,H)**.

After image acquisition, all the copies were transferred to the MP3 server (a Dell R910 server with 512 GB of RAM and two MD1200 storage devices 72 TB). The FLUO images were converted to HSB color space. Pixels having brightness (B) of the HSB color space higher than 25 + c were retained. The constant c is related to the HTS setting. The selected pixels were tagged as foreground to identify the objects using an adapted version of the “combined contour tracing and region labeling” algorithm proposed by Burger and Burge (2008). The objects having an area greater than 1000 pixels and an Euclidean distance lower than 300 to the theoretical pot center were selected. As a result, each plant was represented by one object or “digital plant” from which the area was calculated (Burger and Burge, 2008; Schneider et al., 2012; Camargo et al., 2014). After the FLUO images were resized to 2900 pixels width and proportional height, the pixels in the VIS images were shifted -290 + a pixels in the x-axis and -122 + b pixels in the y-axis coordinates. Then, the positions of the pixels composing the “digital plant” in the FLUO images were used to select the pixels that constitute the “digital plant” in the VIS images. The constant “a” and “b” are parameters related to the HTS deck configuration. The NIR images were processed as the VIS images using 380 to resize and -30 + a, -12 + b to shift the images. The third quartile or 75th percentile of the pixels of every “NIR digital plant” was then calculated (**Figure 2**).

### Color Classification and Clustering

The hue channel of the HSB color space from VIS was equally divided into 65 categories where each pixel of the “digital plant” was classified (color classification; Berger et al., 2012). Each class or category was defined as an interval of intensities and the union of all categories is equal to the full range of the hue channel (0–255). The median hue intensity of each interval with saturation (S) and brightness (B), equal to 255 or its RGB equivalent, was used to identify the class (**Figures 2E,F**). An Euclidean distance matrix of the “digital plants” was then calculated with the the color classes (percentage of pixels) of the VIS images using as input for a hierarchical cluster analysis. The method “ward” of the R function “hclust” was used for clustering. The resulting cluster tree of samples was then divided into two groups using the R function “cutree” (R Core Team, 2013).

### Statistical Analysis

The Cochran-Mantel-Haeszel (“mantelhaen.test” function in R) test was used to assess the effect of the water-limited condition on the survival rate of the lines and the Pearson’s Chi-squared test as a goodness-of-fit test (“chisq.test” function in R) to support the matching between the manual inspection, near infrared and clustering results. The area was assessed using the Student’s *t*-test (“ttest” function in LibreOffice, mode = 2 two tailed test and type = 3 heteroscedastic). The index on the third quartile (Q_3_) NIR intensity was calculated as the Q_3 value_ divided Q_3 base_ multiplied by 100.

### Implementation

The custom image analysis algorithm was developed using java 1.8.0-45^2^ and ImageJ library v1.49 (Rasband, 1997–2012; Schneider et al., 2012). The statistical analysis script was written in R v3.0.2 (R Core Team, 2013) and LibreOffice 4.2.8.2 was used as a complement. PostgreSQL v 9.3.1.was used to construct the database. The analyses were implemented on the MP3 server.

## Results

### Comparison of Protocols

In this study, we have implemented two protocols, namely the “pot protocol” and “pellet protocol,” to assess the non-dependency of our approach to a particular method. The format of the trays, the containers and the soil used in the protocols are completely different (see Materials and Methods). In the first case, the absolute fill capacity was 72.62 (±0.34) g water/pot compared to 30.15 (±0.74) g water/pellet. This is 58% less water available per plant. Under the same environmental conditions, the “pot protocol” took about 30 days to reach 1–2 g water/pot while the “pellet protocol” took about 15 days from 100% fill capacity to 2–3 g water/pallet where visible signs of wilting had appeared triggering the rewatering process (**Figure 1**). In the “pot protocol,” 10 plants failed to germinate against one in the “pellet protocol.” These were removed from the analysis, but the pots and pellet were kept in the trays during the whole experiment.

### Precision Phenotyping

The accuracy of the phenotypes depends largely on the effectiveness of the extraction of the plant digital representation. The Scanalyzer HTS allows overlapping of images from its different sensors. The separation between the background and the plant may become problematic using the VIS, especially at the end of the drought treatment when the colors of the leaves look similar to the soil. The use of the near infrared camera (NIR) has similar difficulties. The shifting of the intensity in the near infrared spectrum due to the change in the water content is proportional to the plants and to the soil making it difficult to separate the foreground from the background without losing information. Fluorescent light illuminated on the leaves has a high level of reflectance compared to the soil where it is null. This feature makes the extraction of the “digital plant” very accurate using the fluorescent light camera (FLUO) as shown in **Figure 2**. However, the occasional algal growth on the surface of the containers with particular soil mixtures may produce a high level of noise. One of the possible solutions is to use mulch as in the case with the “pot protocol.” This was not necessary in the “pellet protocol” since algal growth was not observed. In these two cases, the separation between background and foreground has been easier and more accurate. The FLUO information has then been used to identify the “digital plant” from the images of the other sensors. Hence, the use of the FLUO as a segmentation mask has shown to be the optimal method compared to using a particular method specific to each camera.

### Projected Leaf Area

**FIGURE 3:**
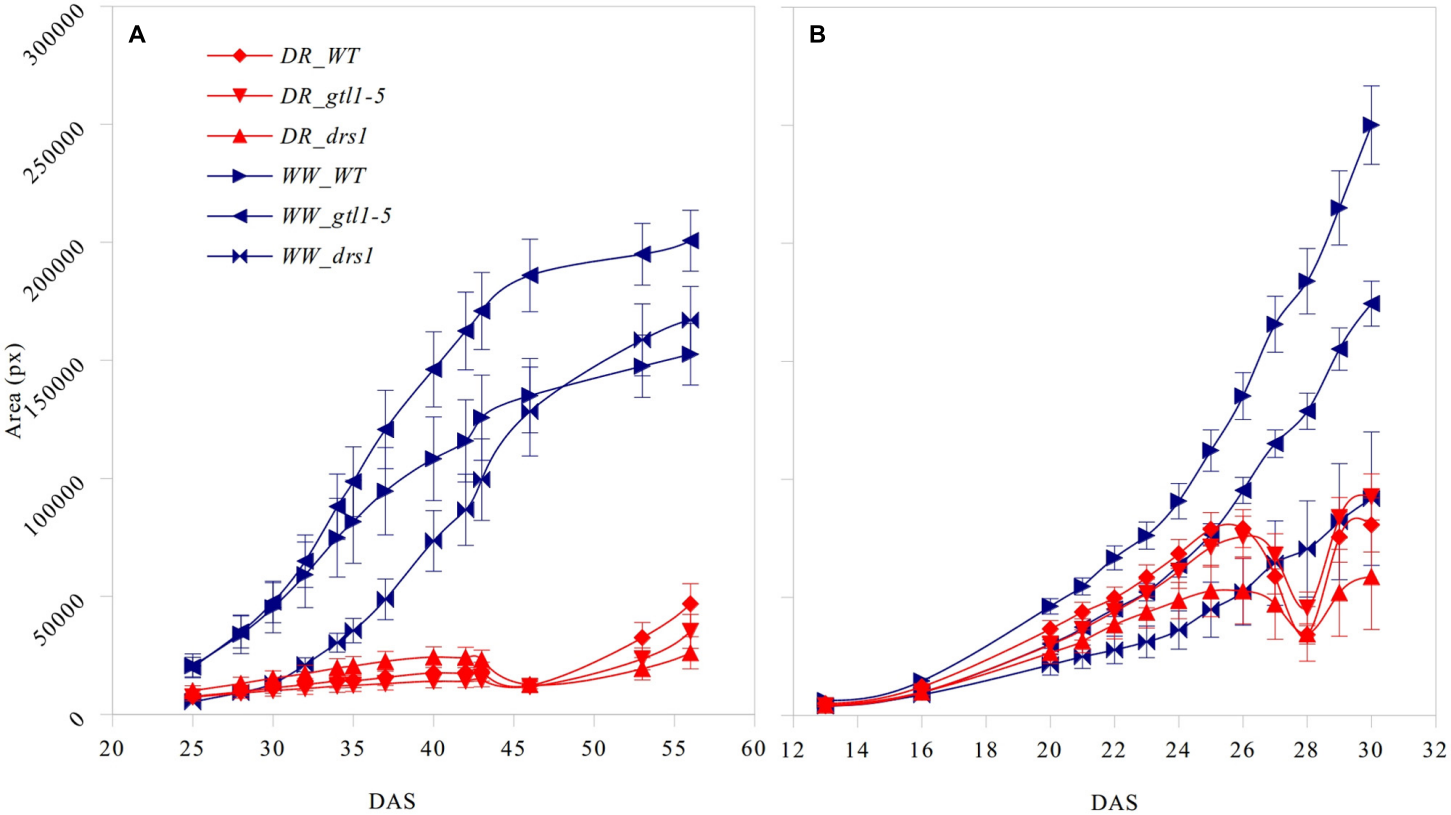
**Projected leaf area.** Number of pixels of the area (mean ± standard error) as a function of the number of days after sowing (DAS) for the three lines in both treatment groups (DR: water-limited or drought and WW: well-watered) for **(A)** “pot protocol” and **(B)** “pellet protocol”.

The projected leaf area showed a clear pattern between both conditions and in both protocols (**Figure 3**). A *p*-value lower than 0.01 was obtained from “days after sowing” (DAS) 27 in the “pellet protocol” and from DAS 28 in the “pot protocol” using a Student’s *t*-test for each measurement. At the end of the water-withholding period, we observed a reduction of the area in the water-limited groups. In the “pellets protocol,” this was observed at DAS 27 where the water content was at 11%: 3.66 g water/pellet (**Figure 3B**). The line *drs1* showed this effect 1 day prior. In the “pot protocol,” the reduction in the area was observed at DAS 46 where the water content was at 2%: 1.31 g water/pot (**Figure 3A**). However, measurements were not taken at DAS 44 and 45. The line *drs1* had also shown a decrease of the area before the others lines at DAS 43. During the recovery period, the area of the water-limited group increased again in both protocols. On average, the water-limited group area was 8% of the well-watered group at the beginning of the recovery phase and reached 20% after 9 days in the “pot protocol,” and 30% at the beginning and 46% after 2 days of recovery in the “pellet protocol.”

### Manual Inspection of Plant Health

In order to evaluate the effectiveness of our method, the samples were classified visually into two categories based on signs of wilting or dryness (Supplementary Tables 1 and 2). In addition, other sources of stress were looked at to verify the correct classification of the data. At the last day of the experiments, “alive” or “dead” status was assigned to each sample as its last classification.

**FIGURE 4:**
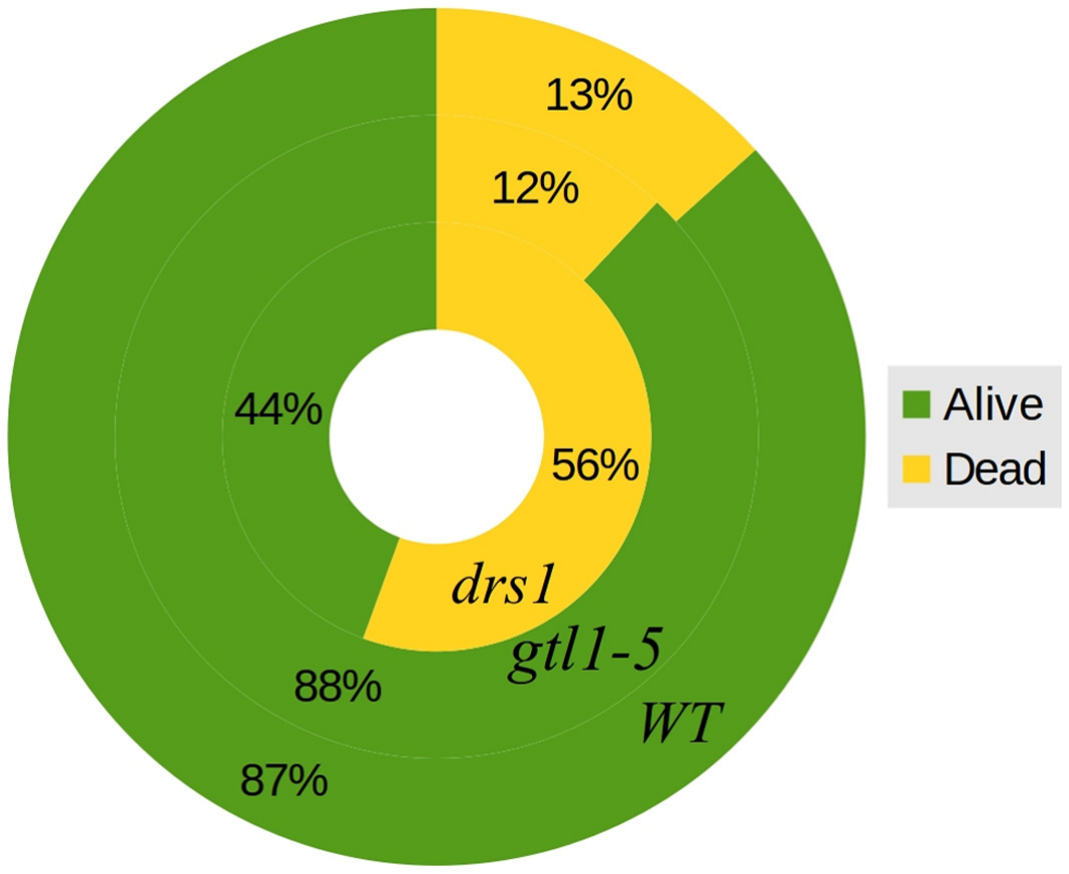
**Survival rates.** General percentages of dead and alive plants per line both protocols together. The outer circle represents WT, the middle circle *gtl1–5* and the inner circle *drs1*.

In the control (well-watered) group of both protocols, none of the plants were classified as wilted or dried. In the “pot protocol,” at DAS 46 (last day of water-withholding), we have observed two of seven WT, two of eight *gtl1–5*, and 6 of 10 *drs1* plants presenting signs of wilting or dryness. Only one WT plant was recovered at the end of the experiment. In the case of the “pellet protocol,” eight of eight WT, six of eight *gtl1–5*, and six of eight *drs1* plants were wilted or dried at DAS 28 (last day of water-withholding). In this case, the recovery rate was higher, seven WT, six *glt1–5*, and two *drs1* plants. In the “pellet protocol,” some of the samples had shown signs of water stress before reaching the end of the drought period, one *drs1* at DAS 25, three *drs1* and one WT at DAS 26 and five *drs1*, two *gtl1–5* and four WT at DAS 27. A Cochran-Mantel-Haeszel test on the water-limited groups on the last day of the recovery periods yielded a p-value lower than 0.01. **Figure 4** shows survival percentages per line.

### Clustering of Color Classes

Here, the samples were clustered using as input the 65 classes of the color classification. Each class is represented by the percentage of pixels of the hue channel of the HSB color space (see Materials and Methods). In this way, the cluster should not be affected by the size of the plant. The resulting tree was then divided into two groups (Supplementary Tables 3 and 4). The expectation is that the samples are classified according to the colors of the leaves. For example, a healthy plant should show a green pattern against the yellow/brown colors exhibited by a dried plant (**Figures 2E,F**).

The efficacy of the clustering was assessed comparing the group assigned by the clustering and the health status from the manual inspection of each plant at every DAS (**Tables 1** and **2**). As expected, before clear signs of dryness, there was no strong agreement in any of the two groups since most of the plants were green without any stress. The minimum percentage of agreement was 40%. At the recovery period, we observed 81, 91, and 91% at DAS 28, 29, and 30 in the “pellet protocol” and 98, 100, and 76% at DAS 46, 53, and 56 in the “pot protocol.” Looking closer at the mismatch at DAS 56, we observed that this is produced by 10 plants in the well-watered group where 80% coincide with the samples to which the inflorescences have been cut. This event has created a visible stress on the plants. The goodness-of-fit test (Pearson’s Chi-squared test) for the “pot protocol” has a *p*-value lower than 0.05 at DAS 25, greater than 0.05 (non-significant) from DAS 28 to 35, a *p*-value lower than 0.05 from DAS 37 to 43, a p-value lower than 1e-10 at DAS 46 and 53, and a *p*-value lower than 1e-03 at DAS 56 (**Table 1**). In the case of the “pellet protocol,” the same test yielded a *p*-value greater than 0.05 (non-significant) at DAS 16 and 23, a *p*-value lower than 0.01 at DAS 13, 20, 21, 22, a *p*-value lower than 1e-04 from DAS 25 to DAS 28, and the most significant *p*-value (<1e-07) at DAS 29 and 30 (**Table 2**).

**Table 1:** Accuracy of the cluster of color classes and the near infrared classifiers compared to the manual inspection during the “pot protocol” experiment.

DAS	25	28	30	32	34	35	37	40	42	43	46	53	56
**Cluster**													
%	64	46	52	48	44	40	68	66	66	34	98	100	76^a^
*p*	4.8e-02	5.7e-01	7.8e-01	7.8e-01	4.0e-01	1.6e-01	1.1e-02	2.4e-02	2.4e-02	2.4e-02	1.1e-11	1.5e-12	2.4e-04
**NIR**													
%	100	100	98	100	100	98	94	98	98	98	94	98	98
*p*	1.5e-12	1.5e-12	1.1e-11	1.5e-12	1.5e-12	1.1e-11	4.9e-10	1.1e-11	1.1e-11	1.1e-11	4.9e-10	1.1e-11	1.1e-11

*p: Pearson’s Chi-squared test *p*-value. ^a^An extra stress in the control group have reduced the percentage of agreement.*

**Table 2:** Accuracy of the cluster of color classes and the near infrared classifiers compared to the manual inspection during the “pellet protocol” experiment.

DAS	13	16	20	21	22	23	24	25	26	27	28	29	30
**Cluster**													
%	83	49	85	74	70	51	79	81	85	83	81	91	91
*p*	6.1e-06	8.8e-01	1.5e-06	7.9e-04	5.6e-03	8.8e-01	8.2e-05	2.3e-05	1.5e-06	6.1e-06	2.3e-05	1.3e-08	1.3e-08
**NIR**													
%	100	100	100	100	100	100	96	89	87	87	94	100	100
*p*	7.1e-12	7.1e-12	7.1e-12	7.1e-12	7.1e-12	7.1e-12	3.6e-10	6.8e-08	3.3e-07	3.3e-07	2.2e-09	7.1e-12	7.1e-12

*p: Pearson’s Chi-squared test *p*-value.*

### Near Infrared Classification

**FIGURE 5:**
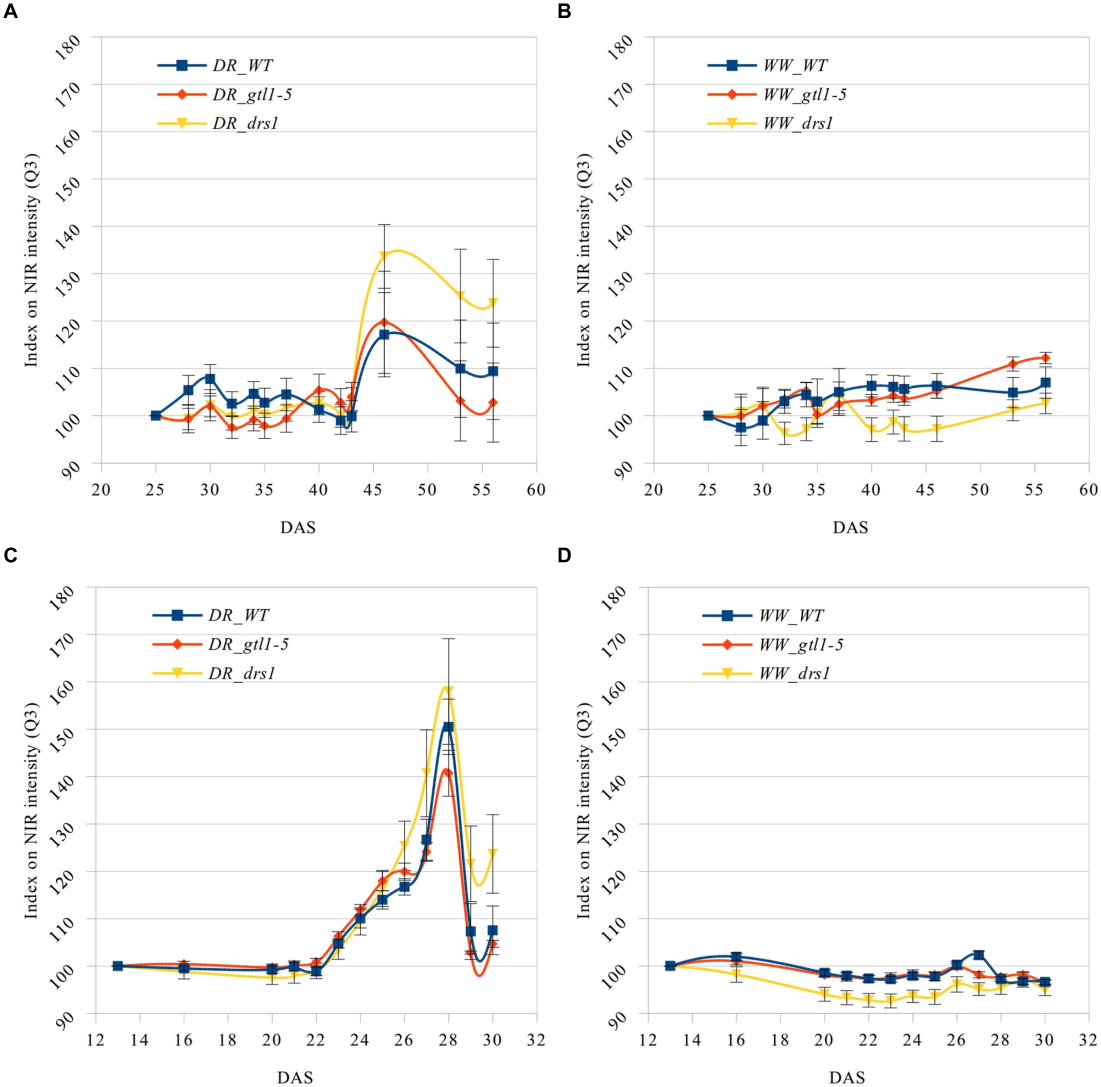
**Q_3_ NIR intensity index.** Means and standard errors of the indexes based on the third quartile (Q_3_) of the NIR intensity for each line and measurement using the first shown DAS as reference. Water-limited **(A,C)** and well-watered **(B,D)** conditions for “pot protocol” **(A,B)** and “pellet protocol” (**C,D**; DR: water-limited or drought and WW: well-watered).

The near infrared has shown a reflectance increase in the water deficit group not exhibited in the control group (**Figure 5**). Here, we have computed the third quartile (Q_3_) or 75th percentile from the pixels of each “NIR digital plant” (Supplementary Tables 5 and 6). Indexes on these values were calculated using DAS 25 and DAS 13 accordingly as references. This allows us to homogenize the start point and to monitor the intensity changes. A condition of the “index base reference” was the homogeneity of their subsequent measurements. In the “pellet protocol,” there were no changes at DAS 16, and only 2% at DAS 20. In the “pot protocol,” we have observed a difference of only 1% at DAS 28 and 2% at DAS 30.

In the last day of withholding water, an increase of about 20% or more in near infrared intensity was registered in both protocols for the samples exposed to water deficit condition (**Figure 5**). The composition of these two groups shows that 100% of the plants having an index higher than 120 in the water-limited group (**Tables 3** and **4**) have presented signs of wilting or dryness in the manual inspection (DAS 53 and 56 for the “pot protocol” and DAS 29 and 30 for “pellet protocol”). The classification of the samples in stress (wilted or dried: index > 120) and non-stress (index < = 120) shows 100% agreement with the manual inspection in all DAS for the three lines in the well-watered group of the “pellet protocol” (**Table 4**). In the case of the “pot protocol,” 100% agreement was observed in all DAS for the lines *gtl1–5* and *drs1*. The WT in this group showed 78% at DAS 37, 89% at DAS 30, 35, 40, 42, 43, 46, 53, and 56, and 100% at DAS 25, 28, 32, and 34 (**Table 3**). As indicated in **Table 3**, 78 and 89% is produced by only three samples at different measurement points. However, they never exhibited an index higher than 127. In the deficit water period, the “pellet protocol” showed 100% agreement from DAS 13 to 23. From DAS 24 to DAS 28 (last water deficit day), the percentage of agreement varies according to the line and DAS with a maximum of 100% and a minimum of 50% (**Table 4**). The plants showing mismatch were classified by the NIR classification as being stressed. While three plants never showed a visible stress phenotype, the other seven presented visible signs of stress at the last day of the water deficit (DAS 28). The “pot protocol” showed 100% agreement in the deficit period except for WT at DAS 37 and 46 and *glt1–5* at DAS 46 (86, 86, and 88%, respectively; **Table 3**). As in the previous case, the two plants not showing visible signs of wilting or dryness have an index higher than 120. We never observed that the NIR classifier pointed to a plant as non-stressed when in reality it was. This may suggest that in some cases the NIR could show a pre-wilting stage of the plant before observed under a visual inspection. In the recovery period of the water-limited group, all lines in all protocols have exhibited 100% agreement between the manual inspection and the NIR classifier even 24 h after rewatering (**Tables 3** and **4**). In this case, a goodness-of-fit test (Pearson’s Chi-squared test) has shown a *p*-value lower than 1e-09 for the “pot protocol” and a *p*-value lower than 1e-06 for the “pellet protocol” in all DAS having a *p*-value lower than 1e-10 in the recovery period of both protocols (**Tables 1** and **2**).

**Table 3:** Agreement between the near infrared classifier and the manual inspection by line and treatment during the “pot protocol” experiment.

DAS	25	28	30	32	34	35	37	40	42	43	46	53	56
**Water-limited**													
*WT* %	100	100	100	100	100	100	86	100	100	100	86	100	100
*gtl1–5* %	100	100	100	100	100	100	100	100	100	100	88	100	100
*drs1* %	100	100	100	100	100	100	100	100	100	100	100	100	100
**Well-watered**													
*WT* %	100	100	89	100	100	89	78	89	89	89	89	89	89
*gtl1–5* %	100	100	100	100	100	100	100	100	100	100	100	100	100
*drs1* %	100	100	100	100	100	100	100	100	100	100	100	100	100

**Table 4:** Agreement between the near infrared classifier and the manual inspection by line and treatment during the “pellet protocol” experiment.

DAS	13	16	20	21	22	23	24	25	26	27	28	29	30
**Water-limited**													
*WT* %	100	100	100	100	100	100	88	88	88	75	100	100	100
*gtl1–5* %	100	100	100	100	100	100	100	63	50	50	75	100	100
*drs1* %	100	100	100	100	100	100	88	88	88	100	88	100	100
**Well-watered**													
*WT* %	100	100	100	100	100	100	100	100	100	100	100	100	100
*gtl1–5* %	100	100	100	100	100	100	100	100	100	100	100	100	100
*drs1* %	100	100	100	100	100	100	100	100	100	100	100	100	100

## Discussion

The aim of this study was to present a comprehensive approach to assess drought stress survival experiments using the benefits of a phenomics platform. One of the advantages of using the HTS at McGill University is the integration of multiple sensor information. Here, this integration was vital to separate the plants from the background using the FLUO to produce a segmentation mask for the images from the other sensors (**Figure 2**; Berger et al., 2010; Klukas et al., 2014). As such, we have provided more power to our analysis than the use of only one sensor. Another advantage is the configuration of the sensors is kept as metadata in the database and, as such, makes each measurement comparable and reproducible.

The two protocols used in this study have been proven to be effective since both have been appropriate to assess the survivability of the mutant lines and the outputs have been consistent. However, the “pellet protocol” was more efficient because it took roughly half of the time to reach the same results (**Figure 1**). This is critical when a large number of lines has to be screened. In addition, the mulch added in the “pot protocol” keeps the moisture in the soil thereby reducing the rate of water loss (Junker et al., 2015), but was necessary to avoid noise in the segmentation process produced by algae growing on the soil surface. Nevertheless, there is no evidence that this part of the protocol has physiological effects (Junker et al., 2015) or affects the experimental output in our hands. Nevertheless, the use of materials that do not have a fluorescence signature such as the Jeffry pellets is recommended over the use of mulch on top of the pots.

The differences in the projected leaf area between both treatment-groups were detected as the water-withholding period progressed (**Figure 3**). These observations are in concordance with previous studies where significant differences in total leaf area of WT (Col-0) were observed between well-watered and water deficit groups (Bouchabke et al., 2008). These differences were significant before the end of the water-withholding period (16 days for the “pot protocol” and 1 day for the “pellet protocol”). This may be explained by the capacity of the soil mixtures to withhold water or by the rate of the drought process. However, we did not observe in the two experiments similar growth patterns for a particular line in any of the groups except for the line *drs1* where the reduction of the area has started earlier compared to the WT and line *gtl1–5*. This may be an indicator of sensitivity to the water-limited condition. The decrease of the projected leaf area is likely a clear sign of a pre-wilting stage and a sensitive line such as the *drs1* may wilt earlier than other lines.

A color trait clustering method has been shown to be a powerful tool as a classifier (Dana and Ivo, 2008). A neural network classifier was also used to successfully identify heat-damaged and green-frost-damaged soybeans (Shatadal and Tan, 2003). The cluster of color classes allowed us to differentiate between the dead and alive plants with an accuracy greater than 90% at the recovery phase using only the percentage of colors (**Tables 1** and **2**). This means that the size of the rosette has not been taken in consideration which is important to avoid a potential bias in the classification. The cluster was always forced to split the samples into two groups. When there was not a visible effect of drought, the division might have revealed an extra source of stress as was the case in the well-watered group of the “pot protocol” (**Table 1**) or might have showed “pre-existent” differences in the plants. However, when the changes of colors were produced as consequence of water deficiency, the cluster classified both groups accurately.

The near infrared light absorption is increased by the presence of water in the leaves (Fahlgren et al., 2015). Previous studies reported a correlation between the near infrared-based indexes and relative water content with severely drought-stressed plants (Berger et al., 2010) as is the case in many survival studies. We have used this property to classify the plants using index numbers based on the third quartile of the NIR pixel distribution. This quartile is more sensitive to an intensity increase than the other two since it is located in the upper part of the “scale.” The accuracy of the NIR classification during the recovery phase for both protocols has been at least 98% (**Tables 1** and **2**). Furthermore, the NIR detected in some cases a pre-wilting stage prior to exhibiting visible signs, it was not affected by the “extra stress” subjected to the inflorescences in the “pot protocol” and it never showed a false negative. Evidently, the performance of the NIR classifier was superior from the beginning in both experiments. In addition, this method is not affected by the size of the rosettes (**Figures 3** and **4**) as is the same case in the cluster of color classes.

The two mutant lines, *dsr1* and *gtl1–5*, were included in our experiments to show the applicability of our method. Our results have shown that the line *drs1* has a significantly lower survival rate compared to WT (**Figure 4**). This is in concordance with the literature as this mutant has been identified as drought sensitive (Lee et al., 2010). *gtl1–5* is a drought resistant line (Yoo et al., 2010). In our case, it showed the same survival rate as WT (**Figure 4**). This may be explained by the high recovery rate of the WT. The rewatering point was based on the observation of lethal effects of dehydration. However, in some cases, the prediction of the recovery of a plant is not evident and should be a good subject for further investigations. The increase of the number of samples with two experimental repetitions using different rewatering points is a possible solution. If the phenomics information is processed in real time, the number of wilted plants could be easily obtained using the NIR classifier to determine exactly these points.

Both classifiers as proxies of the “health status” of the plants have shown to be independent of the size of the rosettes. Passioura (1991) has claimed that plant growth is affected by soil structure in many ways such as the root growth rate or the ability to uptake water and nutrients. This may explain the differences in the projected leaf area ranking of our lines between these protocols. In a survival assay, where a classification of the samples between dead and alive is sought, the projected leaf area is not always a clear index of plant status. Skirycz et al. (2011) pointed that the survivability is not an indicator of growth performance and most of the survival phenotypes in drought are associated with constitutive activation of water-saving mechanisms. Hence, the use of the “NIR” and “the cluster of color classes” classifiers overcomes this limitation of the projected leaf area.

In conclusion, we have shown that our approach is very accurate and can be used with different soil mixtures and containers. The cluster of color classes and the NIR have been shown to be very good classifiers in survival drought experiments. However, the NIR was excellent and efficient during the entirety experiments including the early stages due to its association with water content. When hundreds of samples are tested and analyzed at several time points, the use of a phenomics platform coupled with a bioinformatics approach becomes strictly necessary and this without taking in consideration the objectivity that a human cannot assure.

## Author Contributions

EV, AT, and TB designed the project. EV and TEB wrote the manuscript. EV and AT performed the experiments and the phenotyping analyses. AD selected the mutant lines and assisted with the experimental calibration at beginning of the project. Funding was obtained by TB. All authors discussed the results and provided input on the manuscript.

## Conflict of Interest Statement

The authors declare that the research was conducted in the absence of any commercial or financial relationships that could be construed as a potential conflict of interest.

## Footnotes

1 http://mp3.biol.mcgill.ca

2 www.java.com
