# Supplementary material for: A Comprehensive Approach to Assess Arabidopsis Survival Phenotype in Water-Limited Condition Using a Non-invasive High-Throughput Phenomics Platform
Source: Front Plant Sci. 2015 Dec 15;6:1101. doi: 10.3389/fpls.2015.01101 (PMC4678186; doi:10.3389/fpls.2015.01101)
Supplement: Supplementary file 1 [file Table_1.PDF]

Supplementary Table I. Manual inspection of plant health per sample during the “pot protocol” experiment.

Samples			Manual inspection of plant health												
			DAS												
Sample ID	Line	Treatment	25	28	30	32	34	35	37	40	42	43	46	53	56
WW = well-watered<br>DR = water-limited or drought			Manual inspection<br>Y= wilted or dried<br>N=NON wilted or dried												
1	WT	DR	N	N	N	N	N	N	N	N	N	N	N	N	N
4	WT	DR	N	N	N	N	N	N	N	N	N	N	N	N	N
5	WT	DR	N	N	N	N	N	N	N	N	N	N	Y	N	N
7	WT	DR	N	N	N	N	N	N	N	N	N	N	N	N	N
8	WT	DR	N	N	N	N	N	N	N	N	N	N	N	N	N
25	WT	DR	N	N	N	N	N	N	N	N	N	N	Y	Y	Y
26	WT	DR	N	N	N	N	N	N	N	N	N	N	N	N	N
10	GTL1-5	DR	N	N	N	N	N	N	N	N	N	N	N	N	N
11	GTL1-5	DR	N	N	N	N	N	N	N	N	N	N	N	N	N
13	GTL1-5	DR	N	N	N	N	N	N	N	N	N	N	Y	Y	Y
14	GTL1-5	DR	N	N	N	N	N	N	N	N	N	N	N	N	N
15	GTL1-5	DR	N	N	N	N	N	N	N	N	N	N	N	N	N
16	GTL1-5	DR	N	N	N	N	N	N	N	N	N	N	N	N	N
27	GTL1-5	DR	N	N	N	N	N	N	N	N	N	N	Y	Y	Y
28	GTL1-5	DR	N	N	N	N	N	N	N	N	N	N	N	N	N
17	DRS1	DR	N	N	N	N	N	N	N	N	N	N	Y	Y	Y
18	DRS1	DR	N	N	N	N	N	N	N	N	N	N	Y	Y	Y
19	DRS1	DR	N	N	N	N	N	N	N	N	N	N	N	N	N
20	DRS1	DR	N	N	N	N	N	N	N	N	N	N	Y	Y	Y
21	DRS1	DR	N	N	N	N	N	N	N	N	N	N	N	N	N
22	DRS1	DR	N	N	N	N	N	N	N	N	N	N	Y	Y	Y
23	DRS1	DR	N	N	N	N	N	N	N	N	N	N	Y	Y	Y
24	DRS1	DR	N	N	N	N	N	N	N	N	N	N	N	N	N
29	DRS1	DR	N	N	N	N	N	N	N	N	N	N	Y	Y	Y
30	DRS1	DR	N	N	N	N	N	N	N	N	N	N	N	N	N
1	WT	WW	N	N	N	N	N	N	N	N	N	N	N	N	N
2	WT	WW	N	N	N	N	N	N	N	N	N	N	N	N	N
3	WT	WW	N	N	N	N	N	N	N	N	N	N	N	N	N
4	WT	WW	N	N	N	N	N	N	N	N	N	N	N	N	N
6	WT	WW	N	N	N	N	N	N	N	N	N	N	N	N	N
7	WT	WW	N	N	N	N	N	N	N	N	N	N	N	N	N
8	WT	WW	N	N	N	N	N	N	N	N	N	N	N	N	N
25	WT	WW	N	N	N	N	N	N	N	N	N	N	N	N	N
26	WT	WW	N	N	N	N	N	N	N	N	N	N	N	N	N
9	GTL1-5	WW	N	N	N	N	N	N	N	N	N	N	N	N	N
10	GTL1-5	WW	N	N	N	N	N	N	N	N	N	N	N	N	N
12	GTL1-5	WW	N	N	N	N	N	N	N	N	N	N	N	N	N
13	GTL1-5	WW	N	N	N	N	N	N	N	N	N	N	N	N	N
14	GTL1-5	WW	N	N	N	N	N	N	N	N	N	N	N	N	N
15	GTL1-5	WW	N	N	N	N	N	N	N	N	N	N	N	N	N
16	GTL1-5	WW	N	N	N	N	N	N	N	N	N	N	N	N	N
27	GTL1-5	WW	N	N	N	N	N	N	N	N	N	N	N	N	N
28	GTL1-5	WW	N	N	N	N	N	N	N	N	N	N	N	N	N
17	DRS1	WW	N	N	N	N	N	N	N	N	N	N	N	N	N
19	DRS1	WW	N	N	N	N	N	N	N	N	N	N	N	N	N
20	DRS1	WW	N	N	N	N	N	N	N	N	N	N	N	N	N
21	DRS1	WW	N	N	N	N	N	N	N	N	N	N	N	N	N
22	DRS1	WW	N	N	N	N	N	N	N	N	N	N	N	N	N
24	DRS1	WW	N	N	N	N	N	N	N	N	N	N	N	N	N
30	DRS1	WW	N	N	N	N	N	N	N	N	N	N	N	N	N
