# Supplementary material for: A Comprehensive Approach to Assess Arabidopsis Survival Phenotype in Water-Limited Condition Using a Non-invasive High-Throughput Phenomics Platform
Source: Front Plant Sci. 2015 Dec 15;6:1101. doi: 10.3389/fpls.2015.01101 (PMC4678186; doi:10.3389/fpls.2015.01101)
Supplement: Supplementary file 2 [file Table_2.PDF]

**Supplementary Table II**. Manual inspection of plant health per sample during the "pellet protocol" experiment.

| Samples | | | Manual inspection of plant health | | | | | | | | | | | | |
|---|---|---|---|---|---|---|---|---|---|---|---|---|---|---|---|
| | | | DAS | | | | | | | | | | | | |
| Sample ID | Line | Treatment | 13 | 16 | 20 | 21 | 22 | 23 | 24 | 25 | 26 | 27 | 28 | 29 | 30 |
| WW = well-watered<br>DR = water-limited or drought | | | Manual inspection<br>Y= wilted or dried<br>N=NON wilted or dried | | | | | | | | | | | | |
| 1 | WT | DR | N | N | N | N | N | N | N | N | N | N | Y | N | N |
| 3 | WT | DR | N | N | N | N | N | N | N | N | N | N | Y | N | N |
| 5 | WT | DR | N | N | N | N | N | N | N | N | N | N | Y | N | N |
| 7 | WT | DR | N | N | N | N | N | N | N | N | N | N | Y | N | N |
| 9 | WT | DR | N | N | N | N | N | N | N | N | Y | Y | Y | Y | Y |
| 11 | WT | DR | N | N | N | N | N | N | N | N | N | Y | Y | N | N |
| 13 | WT | DR | N | N | N | N | N | N | N | N | N | Y | Y | N | N |
| 15 | WT | DR | N | N | N | N | N | N | N | N | N | Y | Y | N | N |
| 17 | GTL1-5 | DR | N | N | N | N | N | N | N | N | N | N | N | N | N |
| 19 | GTL1-5 | DR | N | N | N | N | N | N | N | N | N | N | Y | N | N |
| 21 | GTL1-5 | DR | N | N | N | N | N | N | N | N | N | N | Y | N | N |
| 23 | GTL1-5 | DR | N | N | N | N | N | N | N | N | N | N | Y | N | N |
| 25 | GTL1-5 | DR | N | N | N | N | N | N | N | N | N | N | N | N | N |
| 27 | GTL1-5 | DR | N | N | N | N | N | N | N | N | N | Y | Y | N | N |
| 29 | GTL1-5 | DR | N | N | N | N | N | N | N | N | N | N | Y | N | N |
| 31 | GTL1-5 | DR | N | N | N | N | N | N | N | N | N | Y | Y | N | N |
| 33 | DRS1 | DR | N | N | N | N | N | N | N | N | N | N | Y | N | N |
| 35 | DRS1 | DR | N | N | N | N | N | N | N | N | N | N | N | N | N |
| 37 | DRS1 | DR | N | N | N | N | N | N | N | N | N | N | N | N | N |
| 39 | DRS1 | DR | N | N | N | N | N | N | N | N | Y | Y | Y | Y | Y |
| 41 | DRS1 | DR | N | N | N | N | N | N | N | N | N | Y | Y | Y | Y |
| 43 | DRS1 | DR | N | N | N | N | N | N | N | Y | Y | Y | Y | Y | Y |
| 45 | DRS1 | DR | N | N | N | N | N | N | N | N | Y | Y | Y | Y | Y |
| 47 | DRS1 | DR | N | N | N | N | N | N | N | N | N | Y | Y | N | N |
| 2 | WT | WW | N | N | N | N | N | N | N | N | N | N | N | N | N |
| 4 | WT | WW | N | N | N | N | N | N | N | N | N | N | N | N | N |
| 6 | WT | WW | N | N | N | N | N | N | N | N | N | N | N | N | N |
| 8 | WT | WW | N | N | N | N | N | N | N | N | N | N | N | N | N |
| 10 | WT | WW | N | N | N | N | N | N | N | N | N | N | N | N | N |
| 12 | WT | WW | N | N | N | N | N | N | N | N | N | N | N | N | N |
| 14 | WT | WW | N | N | N | N | N | N | N | N | N | N | N | N | N |
| 18 | GTL1-5 | WW | N | N | N | N | N | N | N | N | N | N | N | N | N |
| 20 | GTL1-5 | WW | N | N | N | N | N | N | N | N | N | N | N | N | N |
| 22 | GTL1-5 | WW | N | N | N | N | N | N | N | N | N | N | N | N | N |
| 24 | GTL1-5 | WW | N | N | N | N | N | N | N | N | N | N | N | N | N |
| 26 | GTL1-5 | WW | N | N | N | N | N | N | N | N | N | N | N | N | N |
| 28 | GTL1-5 | WW | N | N | N | N | N | N | N | N | N | N | N | N | N |
| 30 | GTL1-5 | WW | N | N | N | N | N | N | N | N | N | N | N | N | N |
| 32 | GTL1-5 | WW | N | N | N | N | N | N | N | N | N | N | N | N | N |
| 34 | DRS1 | WW | N | N | N | N | N | N | N | N | N | N | N | N | N |
| 36 | DRS1 | WW | N | N | N | N | N | N | N | N | N | N | N | N | N |
| 38 | DRS1 | WW | N | N | N | N | N | N | N | N | N | N | N | N | N |
| 40 | DRS1 | WW | N | N | N | N | N | N | N | N | N | N | N | N | N |
| 42 | DRS1 | WW | N | N | N | N | N | N | N | N | N | N | N | N | N |
| 44 | DRS1 | WW | N | N | N | N | N | N | N | N | N | N | N | N | N |
| 46 | DRS1 | WW | N | N | N | N | N | N | N | N | N | N | N | N | N |
| 48 | DRS1 | WW | N | N | N | N | N | N | N | N | N | N | N | N | N |
