# Supplementary material for: A Comprehensive Approach to Assess Arabidopsis Survival Phenotype in Water-Limited Condition Using a Non-invasive High-Throughput Phenomics Platform
Source: Front Plant Sci. 2015 Dec 15;6:1101. doi: 10.3389/fpls.2015.01101 (PMC4678186; doi:10.3389/fpls.2015.01101)
Supplement: Supplementary file 3 [file Table_3.PDF]

Supplementary Table III. Groups assigned by the cluster of color classes per sample during the "pot protocol" experiment.

Samples			Cluster of percentage of color classes												
			DAS												
Sample ID	Line	Treatment	25	28	30	32	34	35	37	40	42	43	46	53	56
WW = well-watered<br>DR = water-limited or drought			Division of the cluster tree into 2 groups (1 and 2)												
1	WT	DR	1	1	1	1	1	1	1	1	1	1	1	1	1
4	WT	DR	1	1	1	1	1	1	2	2	1	2	2	1	1
5	WT	DR	1	2	1	1	1	1	1	2	1	1	2	1	1
7	WT	DR	2	1	1	1	1	2	1	1	1	2	1	1	1
8	WT	DR	2	2	1	1	1	1	2	2	1	1	1	1	1
25	WT	DR	1	2	1	1	1	1	2	2	1	1	2	2	2
26	WT	DR	1	1	1	1	1	1	1	2	1	1	1	1	1
10	GTL1-5	DR	1	1	1	1	1	1	2	2	1	1	1	1	1
11	GTL1-5	DR	2	1	1	1	1	2	1	1	1	1	1	1	1
13	GTL1-5	DR	1	2	1	1	1	1	2	2	1	1	2	2	2
14	GTL1-5	DR	1	2	1	1	1	1	2	2	1	1	1	1	1
15	GTL1-5	DR	2	2	1	1	1	1	2	1	1	1	1	1	1
16	GTL1-5	DR	2	1	1	1	2	2	1	1	1	2	1	1	1
27	GTL1-5	DR	1	2	1	1	1	1	2	2	1	1	2	2	2
28	GTL1-5	DR	1	2	1	1	1	1	2	2	1	1	1	1	1
17	DRS1	DR	1	2	1	1	1	1	2	2	1	1	2	2	2
18	DRS1	DR	1	2	1	1	1	1	2	2	1	1	2	2	2
19	DRS1	DR	2	1	1	1	2	2	1	1	1	2	1	1	1
20	DRS1	DR	1	2	1	1	1	1	2	2	1	1	2	2	2
21	DRS1	DR	2	2	1	1	1	1	2	2	1	1	1	1	1
22	DRS1	DR	1	2	1	1	1	1	2	2	1	2	2	2	2
23	DRS1	DR	1	2	1	1	1	1	2	2	1	2	2	2	2
24	DRS1	DR	2	1	1	2	2	2	1	1	1	2	1	1	1
29	DRS1	DR	1	2	1	1	1	1	2	2	1	1	2	2	2
30	DRS1	DR	2	1	1	1	2	2	1	1	1	2	1	1	1
1	WT	WW	2	1	2	2	2	2	1	1	2	2	1	1	1
2	WT	WW	2	1	2	2	2	2	1	1	2	2	1	1	2
3	WT	WW	2	1	2	2	2	2	1	1	2	2	1	1	1
4	WT	WW	1	2	2	2	2	2	1	1	1	2	1	1	2
6	WT	WW	1	2	2	2	2	2	1	1	1	2	1	1	2
7	WT	WW	1	2	2	2	2	2	1	1	1	2	1	1	2
8	WT	WW	1	2	1	2	1	1	1	1	1	2	1	1	1
25	WT	WW	1	1	2	2	2	2	1	1	2	2	1	1	2
26	WT	WW	1	1	2	2	2	2	1	1	2	2	1	1	1
9	GTL1-5	WW	1	2	2	2	2	2	1	1	2	2	1	1	1
10	GTL1-5	WW	1	2	2	2	2	2	1	1	1	2	1	1	2
12	GTL1-5	WW	1	2	2	2	2	2	1	1	1	2	1	1	2
13	GTL1-5	WW	1	2	2	2	2	2	1	1	2	2	1	1	1
14	GTL1-5	WW	1	1	2	2	2	2	1	1	2	2	1	1	2
15	GTL1-5	WW	1	2	2	2	2	2	1	1	1	2	1	1	2
16	GTL1-5	WW	1	1	2	2	2	2	1	1	2	2	1	1	2
27	GTL1-5	WW	1	2	2	2	2	2	1	1	2	2	1	1	1
28	GTL1-5	WW	1	2	2	2	2	2	1	1	1	2	1	1	2
17	DRS1	WW	2	1	2	2	2	2	1	1	2	2	1	1	2
19	DRS1	WW	2	1	2	2	2	2	1	1	2	2	1	1	1
20	DRS1	WW	2	1	2	2	2	2	1	1	2	2	1	1	1
21	DRS1	WW	2	1	2	2	2	2	1	1	2	2	1	1	1
22	DRS1	WW	1	1	2	2	2	2	1	1	2	2	1	1	1
24	DRS1	WW	2	1	2	2	2	2	1	1	2	2	1	1	1
30	DRS1	WW	2	2	2	2	2	2	1	1	2	2	1	1	1
