# Supplementary material for: A Comprehensive Approach to Assess Arabidopsis Survival Phenotype in Water-Limited Condition Using a Non-invasive High-Throughput Phenomics Platform
Source: Front Plant Sci. 2015 Dec 15;6:1101. doi: 10.3389/fpls.2015.01101 (PMC4678186; doi:10.3389/fpls.2015.01101)
Supplement: Supplementary file 4 [file Table_4.PDF]

Supplementary Table IV. Groups assigned by the cluster of color classes per sample during the "pellet protocol" experiment.

Samples			Cluster of percentage of color classes												
			DAS												
Sample ID	Line	Treatment	13	16	20	21	22	23	24	25	26	27	28	29	30
WW = well-watered<br>DR = water-limited or drought			Division of the cluster tree into 2 groups (1 and 2)												
1	WT	DR	1	1	1	1	1	1	1	1	1	1	1	1	1
3	WT	DR	1	1	1	1	1	1	1	1	1	1	1	1	1
5	WT	DR	1	2	1	1	1	2	1	1	1	1	2	1	1
7	WT	DR	1	2	1	1	1	2	1	1	1	1	2	1	1
9	WT	DR	1	2	1	1	1	2	1	1	1	2	2	2	2
11	WT	DR	2	2	1	1	1	1	1	1	1	1	2	1	1
13	WT	DR	1	2	1	1	1	2	1	1	1	1	2	1	1
15	WT	DR	1	1	1	1	1	2	1	1	1	1	2	1	1
17	GTL1-5	DR	2	1	1	2	2	1	2	2	2	1	1	1	1
19	GTL1-5	DR	1	2	1	1	1	1	1	1	1	1	1	1	1
21	GTL1-5	DR	1	2	1	1	1	1	1	1	1	1	1	1	1
23	GTL1-5	DR	1	2	1	1	1	2	1	1	1	1	1	1	1
25	GTL1-5	DR	1	2	1	1	1	2	1	1	1	1	1	1	1
27	GTL1-5	DR	1	2	1	1	1	2	1	1	1	1	2	1	1
29	GTL1-5	DR	1	2	1	1	1	1	1	1	1	1	1	1	1
31	GTL1-5	DR	1	2	1	1	1	2	1	1	1	1	2	1	1
33	DRS1	DR	2	1	1	2	2	1	2	2	1	1	2	1	1
35	DRS1	DR	1	1	1	1	1	2	1	1	1	1	1	1	1
37	DRS1	DR	1	2	1	1	1	1	1	1	1	1	1	1	1
39	DRS1	DR	1	1	1	1	2	1	1	1	2	2	2	2	2
41	DRS1	DR	1	1	1	1	1	2	1	1	1	2	2	2	2
43	DRS1	DR	1	1	2	2	2	1	2	2	2	2	2	2	2
45	DRS1	DR	1	1	1	1	1	2	1	1	1	2	2	2	2
47	DRS1	DR	2	1	2	2	2	1	2	2	2	2	2	2	2
2	WT	WW	2	1	1	2	2	1	2	2	1	1	1	1	1
4	WT	WW	1	1	1	1	1	1	1	1	1	1	1	1	1
6	WT	WW	1	2	1	1	1	2	1	1	1	1	1	1	1
8	WT	WW	1	2	1	1	1	2	1	1	1	1	1	1	1
10	WT	WW	1	2	1	1	1	2	1	1	1	1	1	1	1
12	WT	WW	1	2	1	1	1	2	1	1	1	1	1	1	1
14	WT	WW	1	2	1	1	1	2	1	1	1	1	1	1	1
18	GTL1-5	WW	1	1	1	2	2	1	2	2	1	1	1	1	1
20	GTL1-5	WW	1	2	1	1	1	1	1	1	1	1	1	1	1
22	GTL1-5	WW	1	2	1	1	1	2	1	1	1	1	1	1	1
24	GTL1-5	WW	1	2	1	1	1	2	1	1	1	1	1	1	1
26	GTL1-5	WW	1	2	1	1	1	2	1	1	1	1	1	1	1
28	GTL1-5	WW	1	2	1	1	1	2	1	1	1	1	1	1	1
30	GTL1-5	WW	1	1	1	1	1	1	1	1	1	1	1	1	1
32	GTL1-5	WW	1	2	1	1	1	2	1	1	1	1	1	1	1
34	DRS1	WW	1	1	1	1	2	1	1	1	1	1	1	1	1
36	DRS1	WW	2	1	1	1	1	1	1	1	1	1	1	1	1
38	DRS1	WW	2	1	2	2	2	1	2	2	2	2	2	2	2
40	DRS1	WW	1	1	2	2	2	1	2	2	2	2	2	2	2
42	DRS1	WW	1	1	2	2	2	2	1	1	1	1	1	1	1
44	DRS1	WW	1	1	1	2	2	1	1	1	1	1	1	1	1
46	DRS1	WW	1	1	2	2	2	1	2	2	2	2	2	2	2
48	DRS1	WW	2	1	2	2	2	1	2	2	1	1	1	1	1
