# Supplementary material for: A Comprehensive Approach to Assess Arabidopsis Survival Phenotype in Water-Limited Condition Using a Non-invasive High-Throughput Phenomics Platform
Source: Front Plant Sci. 2015 Dec 15;6:1101. doi: 10.3389/fpls.2015.01101 (PMC4678186; doi:10.3389/fpls.2015.01101)
Supplement: Supplementary file 5 [file Table_5.PDF]

Supplementary Table V. Third quartile of the near infrared intensity per sample during the "pot protocol" experiment.

Samples			NIR Intensity – Third quartile (Q_3) or 75th percentile												
			DAS												
Sample ID	Line	Treatment	25	28	30	32	34	35	37	40	42	43	46	53	56
WW = well-watered<br>DR = water-limited or drought															
1	WT	DR	98	102	104	108	109	102	104	105	103	103	105	109	108
4	WT	DR	94	104	105	96	98	110	114	103	102	107	126	103	103
5	WT	DR	97	111	115	98	100	103	104	97	95	95	117	106	105
7	WT	DR	107	124	125	120	123	108	109	110	107	106	108	99	99
8	WT	DR	120	116	119	112	114	108	109	105	101	102	113	96	95
25	WT	DR	97	93	96	94	96	96	98	100	97	98	156	161	160
26	WT	DR	102	102	105	104	107	104	105	101	99	99	104	103	103
10	GTL1-5	DR	112	106	108	105	106	99	100	101	101	101	103	108	107
11	GTL1-5	DR	106	113	115	112	114	111	114	108	107	108	108	107	106
13	GTL1-5	DR	97	94	97	103	104	102	104	116	112	114	172	140	141
14	GTL1-5	DR	101	103	105	99	102	99	101	115	111	112	122	95	94
15	GTL1-5	DR	114	116	119	101	102	105	106	108	106	107	108	94	96
16	GTL1-5	DR	119	129	133	120	122	129	131	130	125	127	125	94	93
27	GTL1-5	DR	110	100	104	100	102	99	100	114	112	114	170	149	146
28	GTL1-5	DR	104	97	100	100	102	100	101	114	110	111	116	96	97
17	DRS1	DR	107	94	97	95	97	99	101	101	99	99	158	170	165
18	DRS1	DR	106	107	111	97	99	102	103	103	100	101	146	161	153
19	DRS1	DR	97	104	102	109	110	101	102	113	112	111	112	92	93
20	DRS1	DR	95	108	112	102	103	112	114	102	99	100	154	152	151
21	DRS1	DR	114	103	104	105	106	101	102	113	110	111	129	100	101
22	DRS1	DR	113	99	102	104	104	105	106	106	104	130	160	158	156
23	DRS1	DR	106	100	102	109	111	109	111	101	99	106	152	142	141
24	DRS1	DR	107	117	125	104	104	104	107	116	114	115	120	99	100
29	DRS1	DR	101	97	100	103	105	105	106	109	105	107	162	149	147
30	DRS1	DR	110	117	121	122	124	117	117	117	111	112	113	94	94
1	WT	WW	88	89	90	90	93	110	112	94	95	95	94	97	101
2	WT	WW	89	91	92	91	91	104	107	91	93	91	91	108	111
3	WT	WW	96	95	93	98	99	98	98	98	97	93	93	97	98
4	WT	WW	105	88	91	92	94	92	93	108	106	106	110	113	113
6	WT	WW	90	94	97	99	100	105	108	93	92	93	94	101	102
7	WT	WW	104	88	89	107	108	91	95	109	108	106	109	97	98
8	WT	WW	95	89	91	110	112	97	98	101	99	100	102	94	96
25	WT	WW	90	108	111	94	94	93	95	112	112	113	113	97	99
26	WT	WW	105	93	94	105	107	90	91	109	111	112	109	96	100
9	GTL1-5	WW	92	105	107	88	90	90	91	97	99	96	97	103	104
10	GTL1-5	WW	90	105	108	94	95	98	99	94	92	94	99	103	103
12	GTL1-5	WW	97	90	92	95	96	91	92	107	108	109	110	114	115
13	GTL1-5	WW	107	89	92	107	111	109	112	111	112	110	110	111	114
14	GTL1-5	WW	93	92	93	96	97	97	101	94	95	95	97	106	106
15	GTL1-5	WW	95	88	90	107	109	97	98	94	95	95	97	105	106
16	GTL1-5	WW	100	92	94	110	112	92	94	98	98	97	98	112	112
27	GTL1-5	WW	91	104	105	92	93	95	99	95	98	98	97	95	98
28	GTL1-5	WW	93	88	90	99	100	90	92	96	97	95	97	102	104
17	DRS1	WW	92	91	92	92	92	102	107	96	97	97	98	99	100
19	DRS1	WW	106	90	91	93	93	94	100	97	99	97	95	110	112
20	DRS1	WW	93	98	98	95	97	93	94	93	95	95	94	94	95
21	DRS1	WW	99	98	99	98	99	107	111	95	95	93	95	99	100
22	DRS1	WW	93	95	97	94	95	94	96	95	97	94	94	96	97
24	DRS1	WW	92	109	110	89	89	94	97	92	94	92	92	95	97
30	DRS1	WW	110	106	108	96	98	102	106	94	96	95	95	98	100
