# Supplementary material for: A Comprehensive Approach to Assess Arabidopsis Survival Phenotype in Water-Limited Condition Using a Non-invasive High-Throughput Phenomics Platform
Source: Front Plant Sci. 2015 Dec 15;6:1101. doi: 10.3389/fpls.2015.01101 (PMC4678186; doi:10.3389/fpls.2015.01101)
Supplement: Supplementary file 6 [file Table_6.PDF]

Supplementary Table VI. Third quartile of the near infrared intensity per sample during the "pellet protocol" experiment.

Samples			NIR Intensity – Third quartile (Q_3) or 75th percentile												
			DAS												
Sample ID	Line	Treatment	13	16	20	21	22	23	24	25	26	27	28	29	30
WW = well-watered<br>DR = water-limited or drought															
1	WT	DR	104	105	105	105	104	109	111	121	123	124	139	105	108
3	WT	DR	100	100	98	97	96	102	105	110	112	113	126	100	102
5	WT	DR	103	100	101	100	99	105	108	113	116	125	151	105	104
7	WT	DR	100	99	96	98	97	103	106	114	116	122	148	101	102
9	WT	DR	99	97	97	98	97	105	111	111	121	152	178	147	142
11	WT	DR	99	98	101	103	102	109	120	125	124	131	153	100	100
13	WT	DR	98	99	97	99	98	103	111	108	110	124	159	101	102
15	WT	DR	98	99	100	100	99	103	109	111	113	123	150	100	101
17	GTL1-5	DR	91	94	93	94	95	102	104	115	117	117	121	94	97
19	GTL1-5	DR	89	88	87	86	87	92	95	104	107	108	123	91	93
21	GTL1-5	DR	87	88	88	87	88	94	100	105	105	110	127	90	92
23	GTL1-5	DR	86	86	87	87	87	91	94	100	103	102	110	89	90
25	GTL1-5	DR	86	86	84	85	85	90	93	96	98	98	104	87	86
27	GTL1-5	DR	87	85	84	86	87	92	98	97	98	112	136	90	92
29	GTL1-5	DR	87	88	88	90	90	93	100	109	108	110	124	89	91
31	GTL1-5	DR	87	88	87	87	87	90	99	100	104	112	140	90	92
33	DRS1	DR	94	93	91	89	90	94	97	105	106	109	128	94	97
35	DRS1	DR	87	85	85	84	86	89	91	98	99	101	112	87	89
37	DRS1	DR	88	88	87	85	85	88	88	91	94	94	95	85	84
39	DRS1	DR	90	87	87	88	88	93	101	107	120	151	170	120	122
41	DRS1	DR	87	86	85	87	88	91	99	98	104	117	144	130	131
43	DRS1	DR	90	85	85	89	90	97	105	115	133	153	171	124	132
45	DRS1	DR	89	85	82	82	83	85	91	96	113	148	170	131	131
47	DRS1	DR	83	90	88	89	90	94	102	113	118	123	129	90	90
2	WT	WW	101	103	101	99	99	99	99	101	104	104	99	99	98
4	WT	WW	96	98	94	93	92	93	94	95	98	98	94	94	93
6	WT	WW	102	101	98	98	97	97	99	97	102	109	97	96	96
8	WT	WW	94	99	93	93	93	92	93	92	93	95	91	92	91
10	WT	WW	93	95	92	92	91	90	90	89	92	94	91	90	90
12	WT	WW	95	95	92	91	91	91	91	91	93	95	91	90	91
14	WT	WW	93	96	94	94	93	93	94	94	94	95	92	91	92
18	GTL1-5	WW	92	93	89	87	87	87	87	88	91	88	88	90	88
20	GTL1-5	WW	88	89	85	83	84	85	84	86	88	86	85	84	84
22	GTL1-5	WW	85	86	83	83	84	84	84	84	86	83	82	84	82
24	GTL1-5	WW	84	85	82	82	81	82	82	80	81	81	81	81	79
26	GTL1-5	WW	83	82	81	82	82	83	83	82	83	82	83	84	82
28	GTL1-5	WW	83	87	83	84	83	84	85	83	85	83	82	81	80
30	GTL1-5	WW	84	82	84	85	83	83	85	84	85	85	84	84	83
32	GTL1-5	WW	87	89	86	84	83	81	83	86	86	85	85	85	83
34	DRS1	WW	90	90	87	85	84	85	85	86	87	88	87	88	86
36	DRS1	WW	86	87	84	81	81	82	83	84	85	84	82	82	80
38	DRS1	WW	89	95	89	91	90	90	90	90	94	91	93	95	91
40	DRS1	WW	91	87	82	82	82	82	83	83	85	84	84	85	84
42	DRS1	WW	91	84	80	80	79	81	82	81	84	84	85	87	87
44	DRS1	WW	90	85	83	83	83	82	83	79	82	82	84	87	85
46	DRS1	WW	88	84	81	81	81	80	81	81	83	82	82	82	80
48	DRS1	WW	90	90	86	84	83	80	82	84	87	85	85	88	86
